# Ovalbumin sensitization and challenge increases the number of lung cells possessing a mesenchymal stromal cell phenotype

**DOI:** 10.1186/1465-9921-11-127

**Published:** 2010-09-21

**Authors:** J Kelley Bentley, Antonia P Popova, Paul D Bozyk, Marisa J Linn, Amy E Baek, Jing Lei, Adam M Goldsmith, Marc B Hershenson

**Affiliations:** 1 Department of Pediatrics and Communicable Diseases, University of Michigan Medical School, 1150 W. Medical Center Dr., Ann Arbor, MI, 48109, USA; 2 Department of Molecular and Integrative Physiology, University of Michigan Medical School, 1150 W. Medical Center Dr., Ann Arbor, MI, 48109, USA; 3 Department of Internal Medicine, University of Michigan Medical School, 1150 W. Medical Center Dr., Ann Arbor, MI, 48109, USA

## Abstract

**Background:**

Recent studies have indicated the presence of multipotent mesenchymal stromal cells (MSCs) in human lung diseases. Excess airway smooth muscle, myofibroblasts and activated fibroblasts have each been noted in asthma, suggesting that mesenchymal progenitor cells play a role in asthma pathogenesis. We therefore sought to determine whether MSCs are present in the lungs of ovalbumin (OVA)-sensitized and challenged mice, a model of allergic airways disease.

**Methods:**

Balb/c mice were sensitized and challenged with PBS or OVA over a 25 day period. Flow cytometry as well as colony forming and differentiation potential were used to analyze the emergence of MSCs along with gene expression studies using immunochemical analyses, quantitative polymerase chain reaction (qPCR), and gene expression beadchips.

**Results:**

A CD45-negative subset of cells expressed Stro-1, Sca-1, CD73 and CD105. Selection for these markers and negative selection against CD45 yielded a population of cells capable of adipogenic, osteogenic and chondrogenic differentiation. Lungs from OVA-treated mice demonstrated a greater average colony forming unit-fibroblast (CFU-F) than control mice. Sorted cells differed from unsorted lung adherent cells, exhibiting a pattern of gene expression nearly identical to bone marrow-derived sorted cells. Finally, cells isolated from the bronchoalveolar lavage of a human asthma patient showed identical patterns of cell surface markers and differentiation potential.

**Conclusions:**

In summary, allergen sensitization and challenge is accompanied by an increase of MSCs resident in the lungs that may regulate inflammatory and fibrotic responses.

## Introduction

Adult bone marrow contains a minority population of mesenchymal stem cells that are thought to contribute to the regeneration of connective tissues such as bone, cartilage, muscle, ligaments, tendons, fat and stroma [1]. These cells demonstrate cell surface markers consistent with a mesenchymal origin (*e.g.*, Stro-1, Sca-1, CD73, CD90, CD105) but fail to express markers associated with a hematopoietic (CD34, CD45) or endothelial cell (CD31) origin. Subsequently, multipotent mesenchymal stem cells were isolated from peripheral sites, including peripheral blood [2], adipose tissue [3], articular synovium [4] and trabecular bone [5]. More recently, it has been suggested that most organs apparently carry their own population of pluripotent mesenchymal stem cells in a perivascular compartment that participate in tissue repair [6]. The perivascular niche has been demonstrated to be the source of mesenchymal stem cells in adipose tissue [7], skeletal muscle [8] and other organs [9].

With regard to the lung, mesenchymal stem cells have been isolated from the bronchial tissue of patients undergoing lobectomy for primary lung tumors [10]. Our laboratory has isolated mesenchymal stem cells from the tracheal aspirates of premature infants undergoing mechanical ventilation for respiratory distress [11]. Mesenchymal stem cells of donor sex identity have been found in lung allografts years after transplantation, suggesting that these stromal cells may originate from the lung tissue itself, perhaps from the perivascular compartment [12]. Finally, we [13] and others [14,15] have shown that, in addition to the above cell types, mesenchymal stem cells may undergo differentiation to myofibroblasts.

Recent work suggests that there is a hierarchy of multipotent mesenchymal stromal cells ranging from true self-renewing stem cells with multilineage differentiation capacity to those with more restricted differentiation potential, until a state of complete restriction to the fibroblast is reached [16]. Since the clonogenicity or self-renewal of most mesenchymal stem cell isolates has not been thoroughly tested, we now refer to cells with the surface markers and differentiation potential of mesenchymal stem cells as mesenchymal stromal cells (MSCs), which have a more restricted differentiation potential.

There is abundant evidence in the literature suggesting that mesenchymal progenitor cells are involved in asthma pathogenesis. Excess airway smooth muscle, caused in part by an increase in the number of smooth muscle cells, has been well-described in asthma [17,18]. Patients with severe asthma also demonstrate an accumulation of myofibroblasts in the airway subepithelium [19-22]. These alterations may extend beyond the central airways to the distal airways and lung parenchyma [23]. Finally, in contrast to control subjects, "activated" fibroblasts have been identified in the bronchoalveolar lavage (BAL) fluid of patients with asthma [24]. We therefore hypothesized that multipotent MSCs, which may serve as progenitor cells of smooth muscle cells and myofibroblasts, are increased in the lungs of ovalbumin-sensitized and -challenged mice, an animal model of allergic airways disease. Previous studies have demonstrated hyperplasia of myofibroblasts in both the large and small airways of these mice, including the terminal bronchioles, alveolar ducts and alveolar walls [25].

## Materials and methods

### OVA-sensitization and challenge

**Figure 1:**
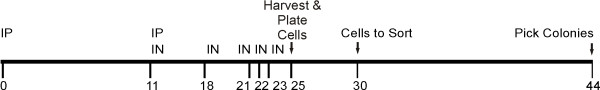
**Schema for OVA sensitization and challenge of mice, isolation of a total lung fibroblast fraction, and sorting of a mesenchymal stromal cell fraction**. Mice were sensitized twice by intraperitoneal (IP) injections at day 0 and day 11 of OVA followed by intranasal (IN) challenges at days 11, 18, 21, 22, and 23. Lungs were isolated, digested with collagenase, and adherent cells plated with daily media changes to remove nonadherent cells. At the end of 5 days, adherent cells were lifted, sorted and, in two additional weeks, processed for colony formation.

Mice (BALB/cByJ, Jackson Labs, Bar Harbor, ME) were sensitized to sterile LPS-free OVA (Pierce, Rockford, IL) or PBS control by intraperitoneal injection and serially challenged over a month with intranasal instillations of OVA or PBS (Figure 1), as previously described [26,27]. This study was approved by the Institutional Animal Care and Use Committee.

### Flow cytometry of whole lung digests and adherent cells

Lungs from PBS- and OVA-treated mice were perfused with 5 mM EDTA, minced into 1-2 mm blocks and digested for 1 hr in 5 mg/ml collagenase. Cells were obtained by filtration through a 70 μm mesh. Red blood cells were lysed with an ammonium chloride buffer. Cells were washed in 10% serum-containing DMEM with 4 mM glucose, 1 mM sodium pyruvate, and 2 mM L-glutamine and plated at 10^6 ^cells/100 mm dish. After 5 days, adherent cells were removed from the plates by trypsin, washed with Ca^2+^, Mg^2+^-containing PBS and blocked with 1% BSA in PBS for 20 min at 4°C. Within three hours after immunostaining (below), cells were sorted using a BD Biosciences FACSDiVa High-Speed Cell Sorter (San Diego, CA) with 350 nm, 488 nm, and 633 nm lasers. Cells were stained with 2.5 μg/mL of FITC- or AlexaFluor (AF)-conjugated antibodies. Typically, anti-Stro-1 was conjugated to AF 350, anti-CD34 was conjugated to FITC, anti-CD105 was coupled to AF555, anti-CD73 was coupled to AF633 and anti-CD45 was conjugated to AF750. Cells were negatively selected for CD34 and CD45 and positively selected for Stro-1, CD73 and CD105 binding (see text). Cells were collected in 1 mL DMEM supplemented with 10% FBS and cultured for 2 weeks to allow colony formation before expansion for study.

### Determination of colony forming unit-fibroblast (CFU-F)

Lungs from PBS- and OVA-treated mice were perfused with 5 mM EDTA, minced into 1-2 mm blocks and digested for 1 hr in 5 mg/ml collagenase. Cells were obtained by filtration through a 70 μm mesh. Trypan blue-excluding cells from each animal were serially diluted and plated in DMEM with 10% FBS at 100 cells/well in 2 mL of medium into a 30 mm well of a 6 well dish (in triplicate). Cells were allowed to grow in 10% FBS/DMEM for 14 days. After 14 days, plates were stained with 0.5% toluidine blue for 5 min, washed three times with PBS, and independent colonies of primary mouse lung fibroblasts were counted, as described previously [28]. A similar procedure was followed to determine the CFU-F of sorted Stro-, CD73, CD105-positive mouse lung MSCs.

### Analysis of cultured sorted cells from the lungs of OVA-sensitized and challenged mice

Cells (passage 3-5 post-sort) were dissociated from plastic using cell dissociation buffer (PBS supplemented with 5 mM EDTA) and analyzed for the surface markers described above, as well as CD90.2 (rat anti-mouse Thy 1.2, clone 30-H12, BD Pharmingen) and Sca-1 (rat anti-mouse Ly 6-A/E, clone e13-161.7, BD Pharmingen). Cells were typically stained with 2 μg/mL FITC- or AF488-conjugated antibodies or isotypic control immunoglobulins and 50 μg/mL propidium iodide.

### Fluorescence microscopy

The pulmonary artery was perfused with EDTA. Mouse lung vasculature was perfused with 5 mM EDTA in PBS injected through the pulmonary artery until the lung blanched. Lungs were inflated to 30 cm H_2_O pressure with 4% paraformaldehyde (Sigma-Aldrich, St. Louis, MO) and placed in formalin overnight. Paraffin-embedded lungs were sectioned with 5 μm-thick sections. AlexaFluor (AF) dye antibody conjugates were prepared using N-hydroxy succinimide esterified dye conjugation reactions with 20 μg antibody and 100 μg activated dye in 10 mM sodium bicarbonate buffer (pH 8.5) for 1 hour. The reaction was quenched by making it 500 mM Tris HCl pH 7.2 for 1 hour, and the conjugated antibody was purified by separating it from quenched dye over a G-50 spin column (GE Healthcare, Piscataway, NJ). Slides were probed with 2 μg/mL AF or fluorescein isothiocyanate (FITC)-conjugated anti-Stro-1 (clone STRO-1, R&D Systems, Minneapolis, MN), anti-CD34 clone MEC14.7, anti-CD45 clone 30-F11, anti-CD73 clone TY/11.8, anti CD105 clone MJ7/18 (each from BioLegend, San Diego, CA) and Cy3- or FITC-conjugated mouse anti-α-smooth muscle actin (clone 1A4, Sigma-Aldrich). Nuclei were visualized with 10 μg/mL Hoechst 33258 (Sigma-Aldrich). Cells were imaged using either an Olympus XI74 microscope (Center Valley, PA) for epifluorescence, a Zeiss Axioplan microscope equipped with an ApoTome and digital AxioCamMR CCD camera, or a Zeiss LSM 150 confocal microscope (Thornwood, NY).

### Differentiation of clonal MSCs

Passage two sorted cells were serially diluted in 96 well plates to 0.3 cells per well. After 2 weeks in this media, individual colonies were trypsinized, dispersed into 100 mm dishes, allowed to grow to confluence and plated on fibronectin-coated slides. Myogenic differentiation was induced by addition of 10 ng/mL transforming growth factor (TGF)-β, 1 μg/mL insulin, 0.55 μg/mL transferrin and 670 ng/mL selenium (Invitrogen, Carlsbad, CA) for 6 days. These cells were immunostained for α-actin (clone 1A4, Sigma-Aldrich) and myosin heavy chain (clone hSM-V, Sigma-Aldrich). Adipogenic differentiation was induced by culture in DMEM supplemented with 5% fetal bovine serum (FBS), 10 μM dexamethasone, 50 μM indomethacin, 100 μM isobutylmethylxanthine (IBMX) and 10 μg/mL insulin for 21 days, as described [11] Formalin-fixed cells were stained for neutral lipids with Oil Red O. Osteogenic differentiation was accomplished by culture in DMEM supplemented with 5% FBS, 100 nM dexamethasone, 10 mM β-glycerophosphate and 50 μg/mL ascorbic acid for 21 days, as described [11]. Formalin-fixed cells were stained for calcium phosphate with alizarin red.

To further assess differentiation state, cells were solubilized and RNA extracted using a guanidine isothiocyanate-phenol protocol (Trizol, InVitrogen). Quantitative two-step real time PCR for differentiation marker transcripts was performed using an Eppendorf realplex2 (Westbury, NY). Transcripts were examined for myocardin (MYOCD, forward sequence, CCCATGGACTCTGCCTATGC; reverse sequence, CTGAGGATCTGTAGCTGCAGGAAT), smooth muscle myosin heavy chain (MHC11, forward sequence, GCCGACACAGCCTACAGAAG; reverse sequence, TTTCCGGCTCCAGACTCA), transgelin (SM22, forward sequence, GCCAACAAGGGTCCATCCTA; reverse sequence, CTCCAGCTCCTCGTCATACTTC), peroxisome proliferator-activated receptor (PPAR)-γ (forward sequence, GGCCCACCAACTTCGGAATC; reverse sequence, GTAAAGGGCTTGATGTCAAAGGAA), fatty acid binding protein 4/adipocyte protein-2 (FABP4, forward sequence, GGCTTTGCCACAAGGAAAGTG; reverse sequence, CGCCCAGTTTGAAGGAAATCTC), osteopontin/secreted phosphoprotein 1 (SSP1, forward sequence, CTCACCATTCGGATGAGTCTGAT; reverse sequence, AGGGACGATTGGAGTGAAAGTG) and osteocalcin/bone γ-carboxyglutamate (gla) protein (BGLAP, forward sequence, CAGCTTGGCCCAGACCTA; reverse sequence, GCCAGGGTCAGCAGAGTG), Expression levels were normalized to that of glyceraldehyde 3-phosphate dehydrogenase (GAPDH; forward sequence, TCCACTCACGGCAAATTCAAC); reverse sequence, CGCTCCTGGAAGATGGTGATG).

### Western analyses

Mouse lung MSCs were washed in PBS, homogenized in 50 mM Tris (pH 7.5), 100 mM NaCl, 50 mM NaF, 40 mM β-glycerophosphate, 2 mM EDTA, 200 μM Na_3_VO_4_, and 1% Triton X-100 containing complete protease inhibitors (Roche Diagnostics, Indianapolis, IN). After centrifugation at 10,000 *g *for 30 min to remove nuclei and cell particles, supernatants were resolved by SDS-PAGE and transferred to nitrocellulose. Membranes were probed with mouse monoclonal anti-α-smooth muscle actin (MAb 1A4; Calbiochem, San Diego, CA), mouse monoclonal anti-myosin heavy chain (MHC) (MAb HSM-V; Sigma-Aldrich, St. Louis, MO) or mouse monoclonal anti-β-actin (Sigma). Antibody binding was detected in Westerns with a peroxidase-conjugated anti-rabbit or anti-mouse IgG and chemiluminescence (Thermo-Pierce, Rockford, IL).

### Gene arrays

We examined the gene expression profile of MSCs using the Illumina MouseWG-6 expression BeadChip platform (San Diego, CA). This system detects 26,766 coding transcripts with well-established annotations, 6,277 coding transcripts with provisional annotations and 56 non-coding transcripts with well-established annotations. Total RNA was extracted using the RNeasy Plus Mini kit (Qiagen, Valencia, CA). Further preparation and analysis was carried out by the University of Michigan Sequencing Core, according to the chip manufacturer's recommended protocol. Hybridized biotinylated cRNA was detected with streptavidin-Cy3 and quantitated using an Illumina BeadArray Reader. The gene expression profile of lung MSCs was compared to primary mouse lung fibroblasts as well as primary mouse bone marrow MSCs, which were isolated from the femurs of OVA-treated mice by flow cytometry, using gates identical to those used for lung MSCs.

For microarray statistical analysis, background-corrected values for each probe on the BeadChip array were extracted using GenomeStudio Data Analysis Software (Illumina). Detection *p*-values were computed using a non-parametric method. Probe signals are ranked relative to signals of negative controls. Detection p-value = 1-R/N, where R is the rank of the gene signal relative to negative controls and N is number of negative controls. Statistical differences in gene expression between lung and bone marrow-derived cells were calculated using the Illumina Custom differential expression algorithm. Multiple tests were corrected by the Benjamini and Hochberg false discovery rate [29]. For qPCR, data were normalized for GAPDH, and differences in gene expression analyzed by paired t test.

### Isolation and characterization of MSCs from the BAL of a patient with asthma

This study was approved by the University of Michigan Institutional Review Board. After informed consent, BAL fluid was obtained from three pediatric asthma patients who were undergoing diagnostic flexible bronchoscopy. Cells were sedimented for 5 min at 500 × g, washed in media, counted, and serial dilutions of 100,000 to 100 cells were plated and allowed to grow for 21 days CellCe, Single colonies were isolated, expanded, and assessed for cell surface markers and pluripotency. Cell surface markers and differentiation potential were compared to human neonatal lung MSCs [11] and primary human lung fibroblasts (Lonza, Walkersville, MD). Cell surface markers were examined by flow cytometry using human-specific antibodies (BioLegend).. Cells were differentiated along adipogenic and osteogenic lines as described above. For chondrogenic differentiation, cells were differentiated along cartilage lines by first sedimenting (10 min at 500 × *g*) 200,000 trypsinized cells in a sterile 15 mL polypropylene culture tube and maintaining them as a pellet for 21 days in serum-free DMEM composed with 10 ng TGF-β1 and 10 ng bone morphogenetic protein-4/mL [30]. At the end of this time cell pellets were fixed overnight in formalin, processed for paraffin embedding and sectioning, and stained for chondroitin sulfate with Alcian blue[31]. A nuclear fast red counterstain was used to help distinguish nuclei acids from acid mucopolysaccharides.

## Results

### OVA sensitization and challenge is associated with the appearance of Stro-1, CD73, and CD105-positive cells with high colony forming potential

**Figure 2:**
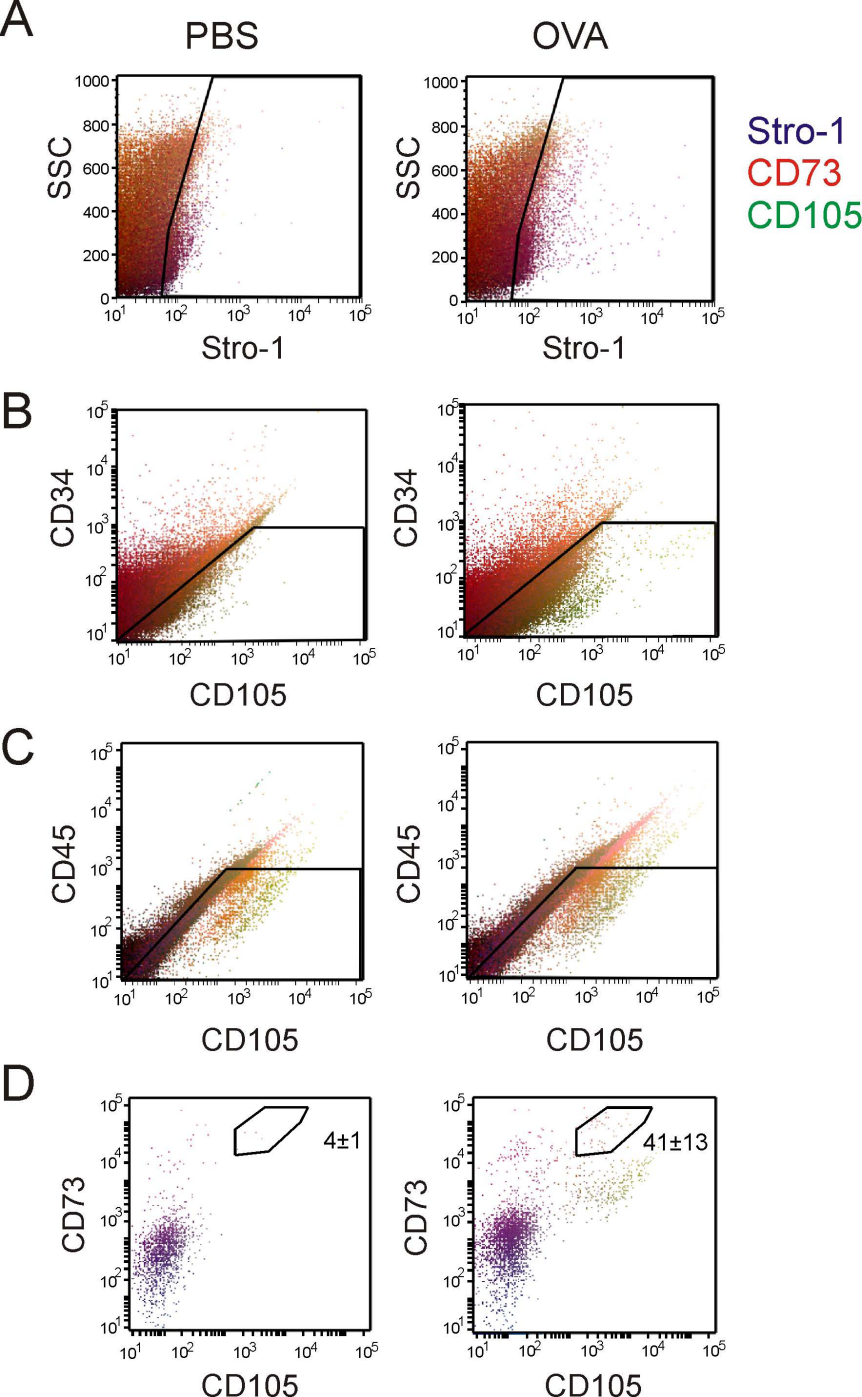
**OVA treatment increases the fraction of mesencymal stromal cell antigen-positive lung cells**. Collagenase dispersible cells were prepared from the lungs of PBS- (left column) or OVA-treated mice (right column). Single cell suspensions from PBS or OVA-treated mouse lungs were allowed to adhere to polystyrene plates for 5 days, trypsinized, blocked with 1% BSA and incubated with AF350-conjugated anti-Stro-1, AF488 anti-CD34, AF750 anti-CD45, AF555 anti-CD105, and AF633 anti-CD73. Data from 100,000 events are shown, with Stro-1 fluorescence intensity in blue, CD73 in red and CD105 in green. Gates were drawn to select a Stro-1-positive (A), low CD34 (B) and low CD45 population (C). In panel D, the gates indicated in panels A, B and C were used to produce the CD73 vs. CD105 plots shown (dot size is adjusted to make the lower cell numbers visible). The average number of cells in the various CD73-, CD105-positive subpopulations shown is provided Table 1. There was a significant increase in the number of CD34-, CD45-negative, CD73-, CD105-positive cells in lungs from OVA-treated mice.

We used flow cytometry to examine cells for cell surface markers typically associated with MSCs (Figure 2). Cells from whole lung digests were plated on plastic for five days to enrich for adherent lung fibroblast cells. We stained live cells from PBS- and OVA-treated mice for Stro-1, CD73, CD105, CD34 and CD45. We found a subpopulation of fibroblast-like cells from OVA-treated mice which showed greater Stro-1 expression (Figure 2A). We found distinct side populations of cells from OVA-treated mice which showed greater CD105 expression (Figures 2B and 2C), Both populations gated in the same forward scattering area. To eliminate hematopoietic cells, CD34 and CD45-positive cells were delineated by the windows shown (Figures 2B and 2C) and further gated for CD73 and CD105 expression, leaving a population of Stro-1-, CD73- and CD105-positive cells (Figure 2D). This population represents about 0.04% of the original lung cell digest (Table 1) and is here designated a lung MSC population. These cells were rarely detectable in PBS-treated lungs (0.004%).

**Table 1:** Populations of Stro-1-, CD105-positive, CD34-CD45-negative cells from PBS- and OVA-treated lungs.

*Population of Cells Analysed*	*PBS*	*OVA*
*Total population*	100,000	100,000
*High Stro-1, low CD34, low CD45, low CD73, high CD105*	14 ± 7	36 ± 19
*High Stro-1, low CD34, low CD45, medium CD73, high CD105*	12 ± 6	138 ± 61
*High Stro-1, low CD34, low CD45, high CD73, high CD105*	4 ± 1	41 ± 13*

Cells obtained and sorted as described above were counted from 3 sets of 5 PBS- or OVA-treated mice (*P < 0.05, unpaired t-test).

The potential of adherent cells to form colonies was assessed by colony-forming unit-fibroblast (CFU-F) assay [28]. Sorted cells and primary mouse lung fibroblasts were serially diluted and 100 cells were plated in DMEM with 10% FBS. After 14 days, plates were fixed and stained. Sorted cells demonstrated a CFU-F of 29.0 ± 1.4 (mean ± SEM, n = 4) out of 100 cells plated, significantly greater than unsorted lung cells from either PBS (1.5 ± 0.1)- or OVA-treated (8.9 ± 2.2) mice (n = 6 for both groups, *P *< 0.05, ANOVA).

**Figure 3:**
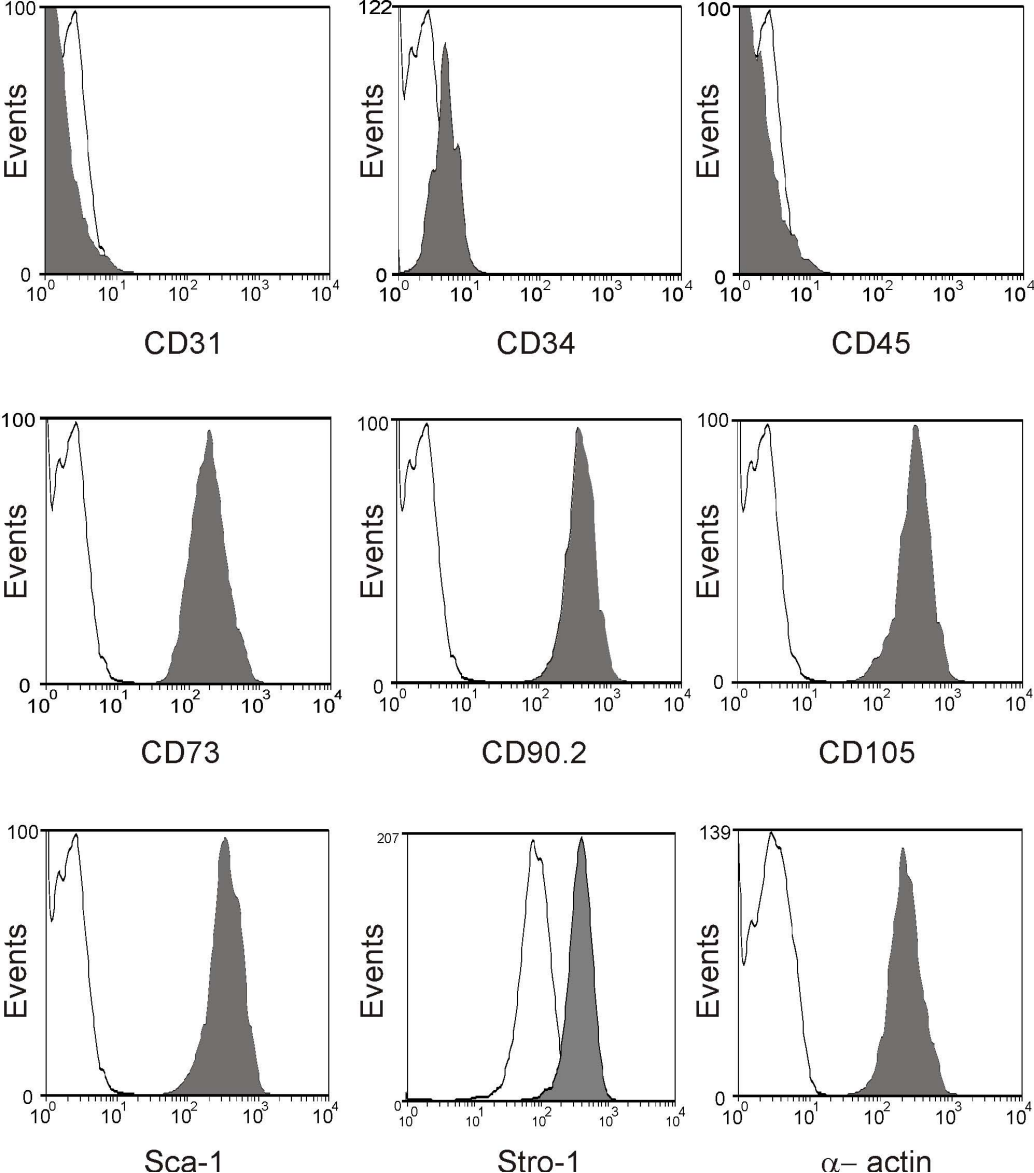
**Sorted cells retain cell surface markers typically associated with multipotent stromal cells**. The CD34-, CD45-negative, Stro-1-, CD73-, CD105-positive cell fraction was plated, grown to confluence, passaged three times, removed with cell dissociation buffer, and bound to either AF488-conjugated control Ig (open curve) or antibody to the indicated antigen (grey curve). Cells in G_0_/G_1 _phase were gated by staining with propidium iodide. Event number is indicated on the y-axis.

Sorted and clonal cells were trypsinized after two further passages and processed for flow cytometry using the same antibodies with which they were initially sorted. The sorted cells retained MSC cell surface antigens after propagation, and also stained positively for CD90.2 and Sca-1 (Figure 3). In the mouse lung, the CD45-, CD31-negative, Sca-1-positive phenotype has been associated with the mesenchymal progenitor cell lineage [32]

**Figure 4:**
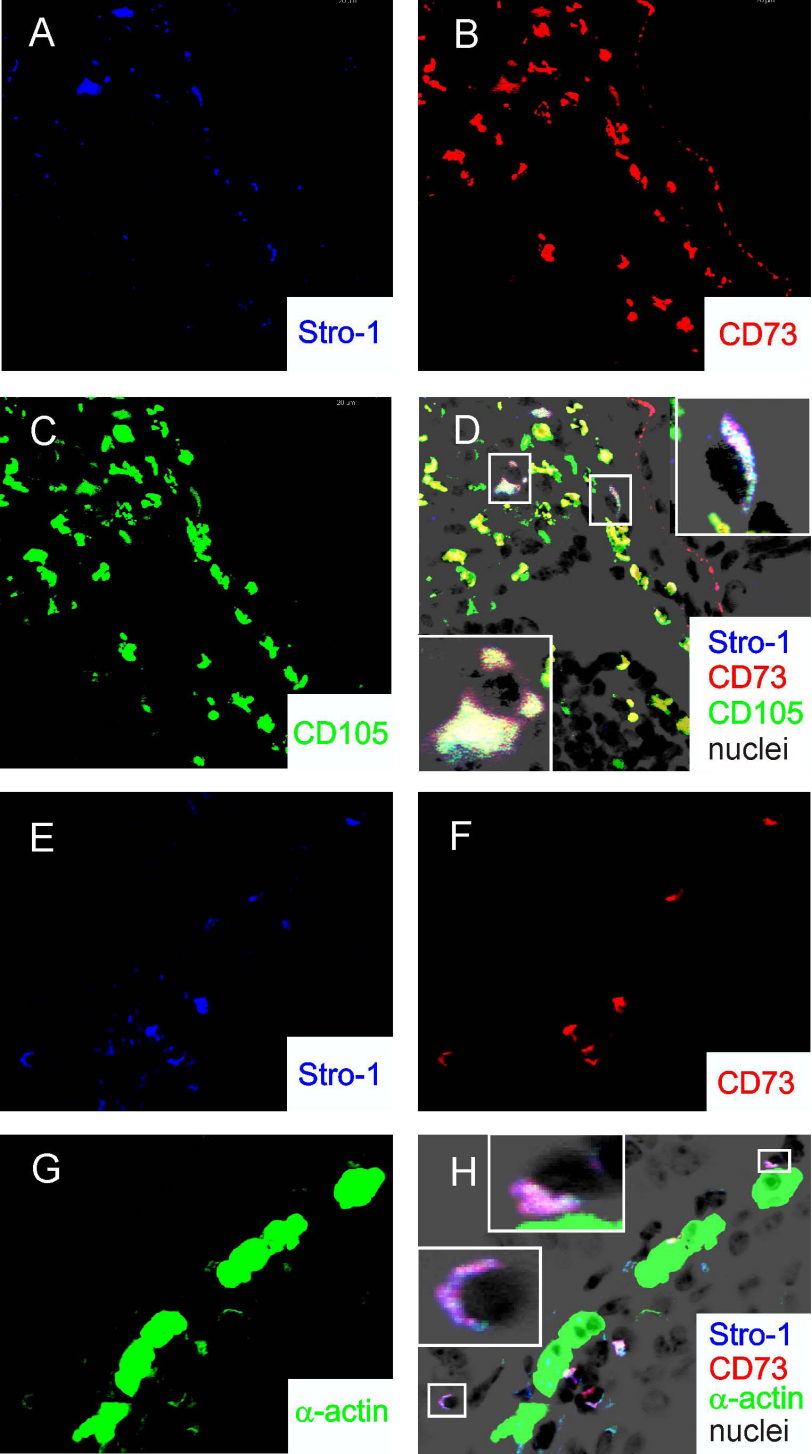
**Presence of Stro-1-, CD73-, CD105-positive cells in the lungs of OVA-treated mice**. A-D. Sections from OVA-sensitized and -challenged mouse lungs (at 630× original magnification) were stained for nuclei with Hoescht 33258. These are shown as a negative grayscale image in black. AF633-conjugated anti Stro-1 is shown in blue (A), AF555-conjugated anti-CD73 is shown in red (B) and AF488-conjugated anti-CD105 is shown in green (C). In D, the merged image shows co-localization of CD73 and CD105 (yellow/orange) and Stro-1, CD73 and CD105 (white). The insets show isolated cells at higher magnification. (There is some non-specific crossreactivity with the epithelial basement membrane in the red channel.) E-H. Lung sections from OVA-treated mice were stained for nuclei with Hoechst 33258 (black), AF633-conjugated anti-Stro-1 (blue, E), AF555-conjugated anti-CD73 (red, F) and AF488-conjugated anti-α-smooth muscle actin (green, G). In H, the merged image shows co-localization of Stro-1 and CD73 (magenta), Stro-1 and α-actin (turquoise) and Stro-1, CD73 and α-actin (white). The insets show isolated cells at higher magnification. Some positive cells lie adjacent to the airway smooth muscle layer.

We examined sections from PBS- and OVA-treated mouse lungs for surface markers typically associated with MSCs including Stro-1, CD73 and CD105. In humans, Stro-1 is almost exclusively found on bone marrow-derived stromal cells [33]. Stro-1 localization has also been noted in mouse mesenchymal cells of the bone marrow [34] and developing periodontium [35]. CD73 (SH3/SH4) and CD105 (SH2/endoglin) are localized at the plasma membrane of bone marrow mesenchymal stem cells [36]. A small number of peribronchial cells stained positively for Stro-1, CD73, CD105 and α-smooth muscle actin (Figure 4).

### Sorted Stro-1(+), CD73(+), CD105(+) cells may be differentiated along a variety of mesenchymal lineages

**Figure 5:**
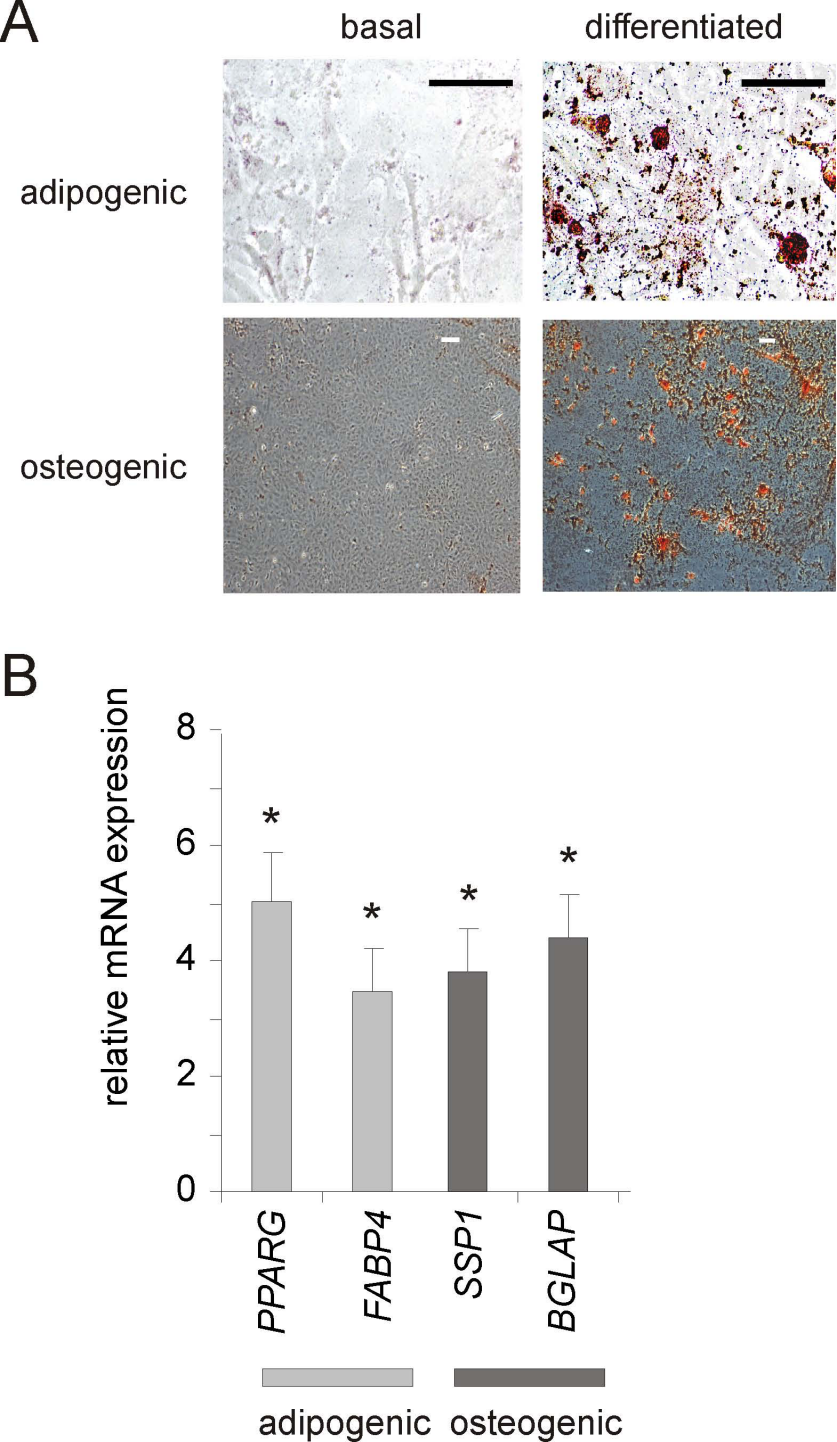
**Sorted Stro-1(+), CD73(+), CD105(+) cells may be differentiated along adipogenic and osteogenic lines**. In panel A, cells were differentiated for 21 days as described in the text. Adipogenic differentiation was monitored by staining for neutral lipids with Oil Red-O (stains red). Osteogenic differentiation was assessed by staining of calcium phosphate with alizarin red (red). B. Four different single cell clones isolated from different sorting experiments were exposed to adipogenic and osteogenic media (see text). Bars depict the fold increase in gene expression by quantitative PCR (mean ± SEM, *p < 0.05, paired t test).

**Figure 6:**
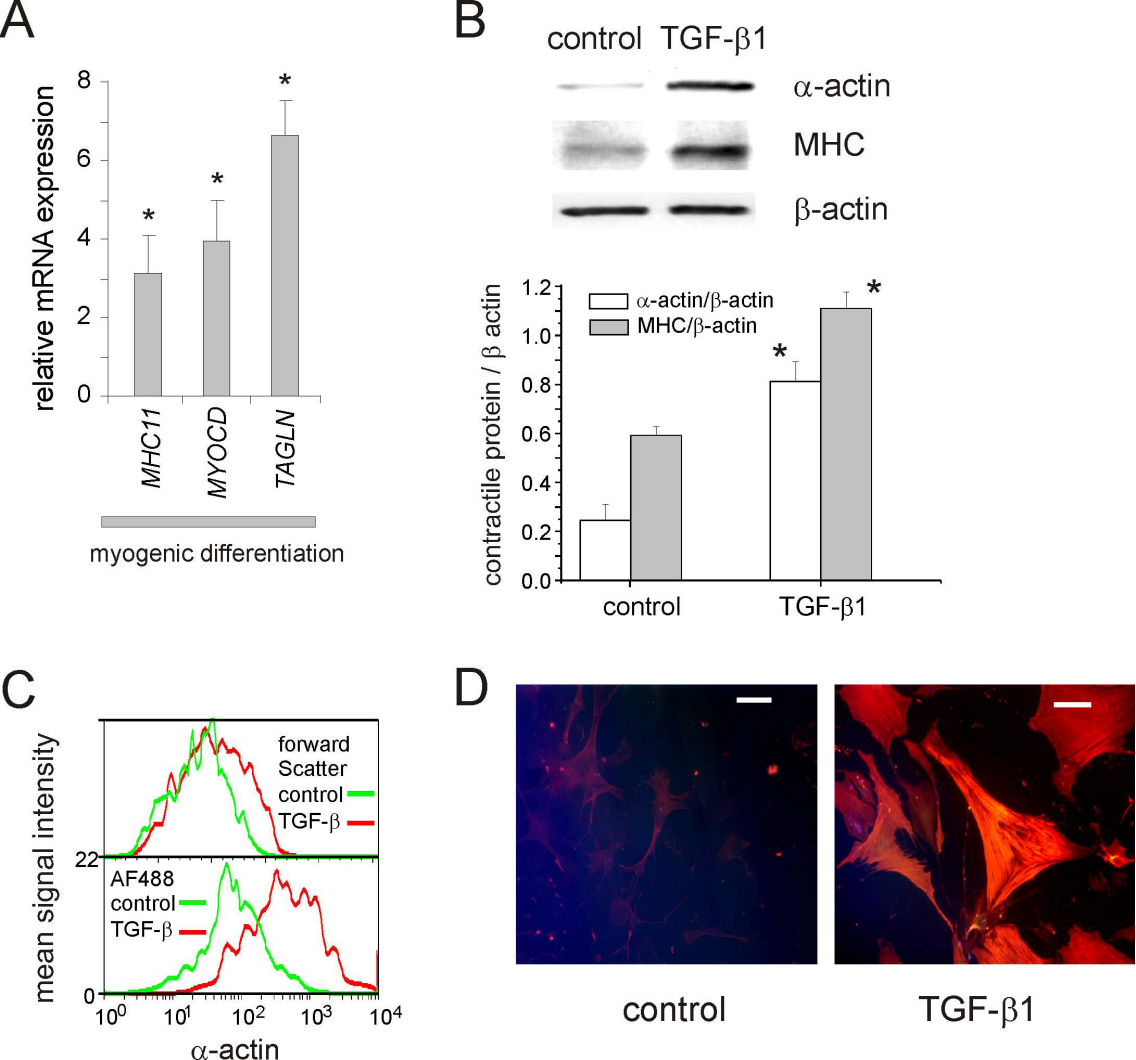
**TGF-β induces myofibroblastic differentiation of lung MSCs**. In panel A, three different clonal cell lines were incubated for 6 days under serum-free conditions without or with 10 ng TGF-β/mL. A. qPCR reactions were run on the cDNA produced from RNA from the cells assessing the relative expression of myosin heavy chain 11 (*MHC11*), transgelin (*TAGLN*), or myocardin (*MYOCD*). Expression of all three was significantly elevated with TGF-β treatment (N = 3, p < 0.05). B. Western analyses demonstrated increases in α-actin and myosin heavy chain (MHC) synthesis. Signals were quantified by densitometry and normalized to β-actin (N = 3, p < 0.05). C. TGF-β increases cell size and α-smooth muscle actin expression. Forward scatter (top panel) and α-actin immunostaining (bottom panel) for cells serum deprived for 6 days (green) or treated with TGF-β (10 ng/ml) for 6 days (orange). D. TGF-β produces an increase in cell size, α-actin, and MHC immunostaining. Myofibroblastic differentiation was assessed by staining with AF488-conjugated anti-MHC (green) and anti α-actin-Cy3 conjugate (red). Co localization of α-actin and MHC Is shown In yellow to orange. White bars indicate 100 μm.

Cells from Stro-1-, CD73-, CD105-positive, CD34-, CD45-negative subpopulations were seeded onto plastic dishes. Only the high CD73, high CD105 cells grew well and were studied further. To isolate individual clones, passage two sorted cells were serially diluted in 96 well plates to 0.3 cells per well. After two weeks of growth in this media, wells with cells were trypsinized. Cells were dispersed into 100 mm dishes, allowed to grow to confluence, plated on fibronectin-coated slides and differentiated along adipogenic, osteogenic and myofibroblast lines (Figures 5, 6). When cells are treated with adipogenic medium (10 μM dexamethasone, 100 μM IBMX, 50 μM indomethacin and 100 μg/mL insulin), cells accumulated neutral lipid vesicles (Figure 5A) and expressed more mRNA encoding *PPARG *and *FABP4 *(Figure 5B). Osteogenic differentiation with 0.1 μM dexamethasone, 10 mM β-glycerophosphate and 50 μg/ml ascorbic acid caused cells to increase calcium phosphate deposition as well as mRNA expression of *SSP1 *and *BGLAP*. TGF-β treatment induced expression of *MYOCD*, *MHC11*, and *TAGLN *(Figure 6A). Western analyses of these cells demonstrated significantly more α-actin and MHC (Figure 6B). The size of α-actin-positive cells was increased, as assessed by increased forward scatter (Figure 6C). When cells were treated with TGF-β, many differentiated into filamentous α-actin- and MHC-expressing myofibroblasts (Figure 6D). On the basis of their cell surface markers, ability form colonies, and differentiation potential, these cells meet established criteria for MSCs [37].

### Sorted lung MSCs have a significantly different gene expression pattern from either unsorted lung fibroblasts

**Figure 7:**
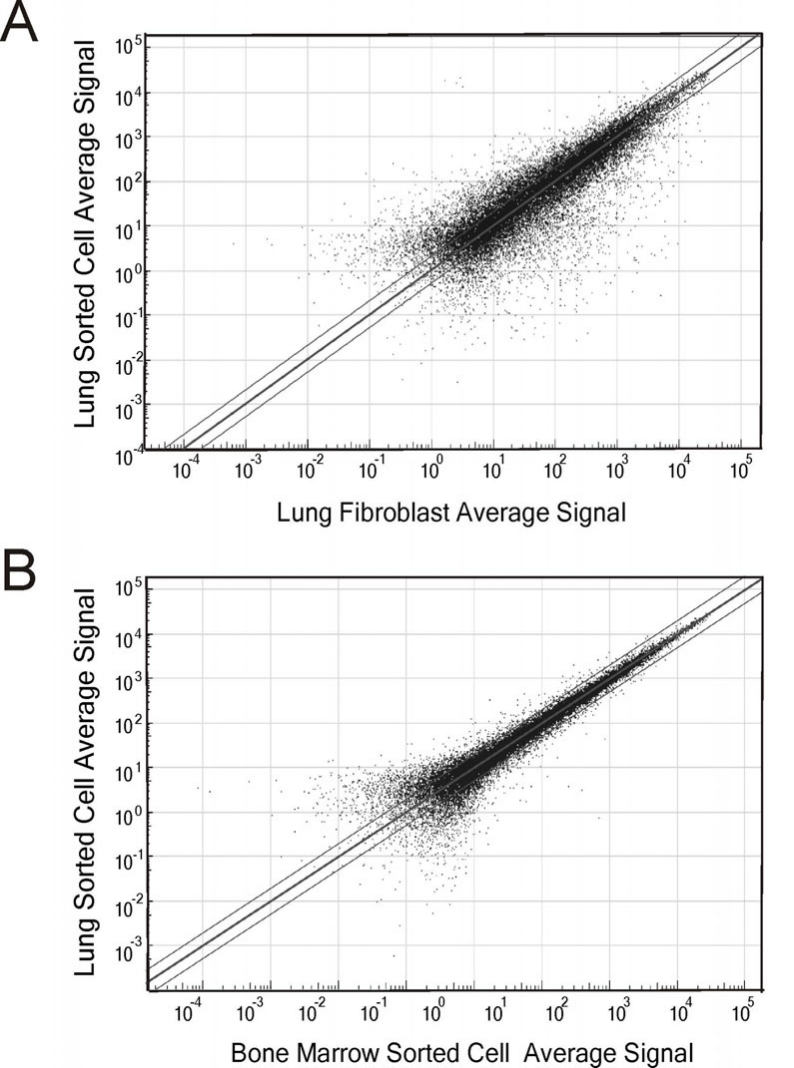
**Concordance of gene probe detection p-values between sorted lung multipotent stromal cells, lung fibroblasts (A) and bone marrow sorted cells (B)**. Parallel lines signify 1.5 fold change based on average probe signal. There was not a high degree of concordance between lung MSCs and lung fibroblasts, suggesting that lung multipotent stromal cells represent a unique population of cells in the lung.

To better characterize the differences between sorted lung MSCs and other resident fibroblast-like cells, mRNA samples from three different sorted clonal lines were used to analyse patterns of gene expression. Out of 33043 mouse mRNA transcripts, a detection p ≤ 0.05 for 12,331 genes of mouse lung MSCs was obtained. Global transcript expression concordance (r^2^) between the sorted lung MSCs and adherent unsorted lung fibroblasts was 0.7539 (Figure 7A). We found a differential p value of ≤0.05 for 5559 genes between sorted mouse lung MSCs and unsorted adherent lung fibroblasts. On the other hand, there was high concordance between lung and bone marrow MSCs (r^2 ^= 0.9872) (Figure 7B). Thus, the sorted multipotent stromal cells represent a distinct subpopulation of cells within the lung.

Of the 5559 genes differing between lung MSCs and lung fibroblasts, 2480 were upregulated. These genes included selected cytokines and paracrine factors; receptors, signaling intermediates and transcriptional regulators of the TGF-β1 and TNF-α pathways; and receptor tyrosine kinases and their downstream signaling intermediates (Table 2 and Additional File 1, Table S1, supplement).

**Table 2:** Selected genes upregulated in sorted lung MSCs relative to lung fibroblasts.

*Symbol average*	*Lung MSCs average*	*Lung fibroblast*	*Fold difference*	*Differential p-value*
*Secreted cytokines and paracrine factors*				
*Prl2c3*	20800	2.91	7147.77	1.00E-13
*Prl2c4*	16700	2.49	6706.83	1.01E-13
*Prl2c2*	16400	2.37	6919.83	1.01E-13
*Efnb2*	250	42.4	5.90	2.98E-08
*Prl2c5*	224	71.5	3.13	7.15E-08
*Grem1*	17700	2520	7.02	1.42E-06
*Ptges3*	2080	763	2.73	1.20E-05
*Fgf10*	3710	853	4.35	0.000233
*Ctf1*	265	109	2.43	0.000226
*Ccl25*	171	58.9	2.90	0.000239
*Fgf7*	7600	3580	2.12	0.00038
*Efna4*	176	85.3	2.06	0.00268
*Bmper*	2060	1020	2.02	0.0041
*Lefty1*	411	244	1.68	0.0163
*Gdf11*	52.3	7.81	6.70	0.0279
*Thbs1*	961	300	3.20	0.0293
*Cx3cl1*	384	81.6	4.71	0.0311
*Sema3a*	638	14.8	43.11	0.0367
*Spp1*	12500	8920	1.40	0.0469
*TGF-β1 signaling*				
*Smad2*	334	86.4	3.87	5.67E-07
*Smad3*	889	410	2.17	5.67E-07
*Mapk13*	387	104	3.72	1.28E-06
*Hdac2*	2440	1300	1.88	1.58E-05
*Trrap*	207	54.2	3.82	4.02E-05
*Hdac4*	179	69.6	2.57	0.000433
*Mapk11*	115	30.3	3.80	0.000661
*Tgfbrap1*	603	195	3.09	0.00172
*Nfatc3*	287	121	2.37	0.00226
*Hdac3*	1080	377	2.86	0.00241
*Hdac10*	60.3	19.4	3.11	0.00378
*Acvr2b*	177	107	1.65	0.0308
*Endogl1*	77.5	39.1	1.98	0.0462

Abbreviations are: *Prl2c3*, encoding prolactin family 2, subfamily c, member 3; *Prl2c4*, prolactin family 2, subfamily c, member 4 *; Prl2c2. *prolactin family 2, subfamily c, member 2; *Efnb2*, ephrin B2; *Prl2c5*, prolactin family 2, subfamily c, member 5; *Grem1*, gremlin 1; *Ptges3*, prostaglandin E synthase 3; *Fgf10*, fibroblast growth factor 10; *Ctf1*, cardiotrophin 1; *Ccl25*, chemokine (C-C motif) ligand 25; *Fgf7*, fibroblast growth factor 7 or keratinocyte growth factor; *Efna4*, ephrin A4; *Bmper*, BMP-binding endothelial regulator; *Lefty1*, left right determination factor 1; *Gdf11*, growth differentiation factor 11 (BMP 11); *Thbs1*, thrombospondin 1; *Cx3cl1*, chemokine (C-X3-C motif) ligand 1; *Sema3a*, sema domain, secreted, (semaphorin) 3A; *Spp1*, secreted phosphoprotein 1, osteopontin; *Smad2*, MAD homolog 2 (Drosophila); *Smad3*, MAD homolog 3 (Drosophila); *Mapk13*, mitogen activated protein kinase 13 (p38); *Hdac2*, histone deacetylase 2; *Trrap*, transformation/transcription domain-associated protein; *Hdac4*, histone deacetylase 4; Mapk11, mitogen-activated protein kinase 11 (p38 δ); *Tgfbrap1*, transforming growth factor, beta receptor associated protein 1; *Nfatc3*, nuclear factor of activated T-cells, cytoplasmic, calcineurin-dependent 3; *Hdac3*, histone deacetylase 3; *Hdac10*, histone deacetylase 10; *Acvr2b*, activin receptor IIB; *Endogl1*, endoglin (CD105);

### Isolation of mesenchymal progenitor cells with surface markers of MSCs from a patient with asthma

**Figure 8:**
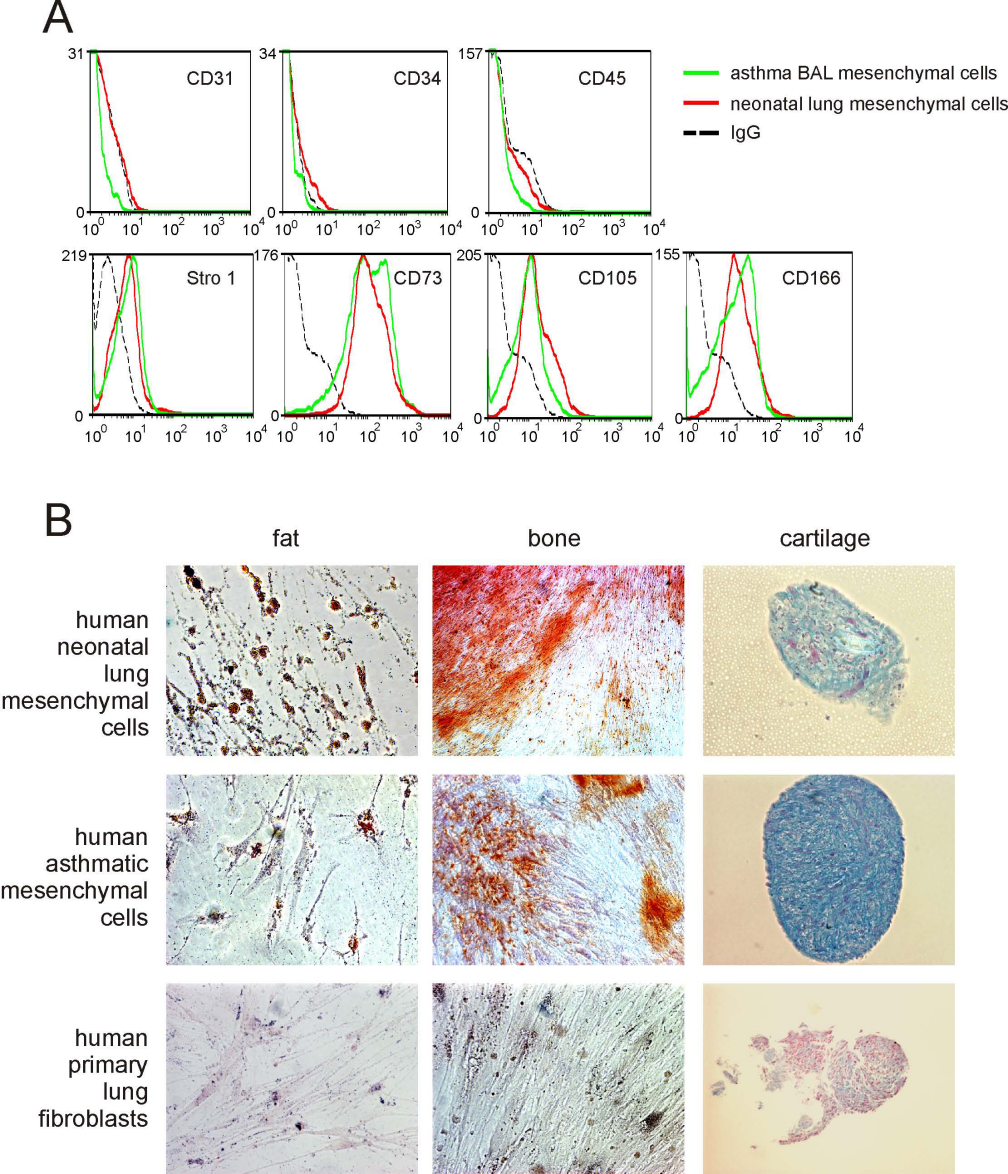
**Isolation of a mesenchymal progenitor cells from the BAL of a patient with asthma**. A. Cells are negative for CD31, CD34 and CD45, and positive for Stro-1, CD73, CD105 and CD166. Mesenchymal cells from a patient with asthma (green) are compared with neonatal lung mesenchymal cells (red). IgG control is shown in black. B. Colonies of cells derived from the tracheal aspirates of human premature infants and a patient with asthma were isolated, grown and treated for differentiation along fat, bone or cartilage lines. Responses were compared with primary human lung fibroblasts. Shown are oil red o staining for fat droplets (left column of panels), alizarin red staining for calcium phosphate (middle column of panels) or alcian blue staining for acid mucopolysaccharide sulfates (nuclear fast red counterstain).

We have isolated pluripotent mesenchymal cells from the tracheal aspirates of premature infants undergoing mechanical ventilation for respiratory distress [11]. Activated fibroblasts have previously been isolated from human asthma patients [24]. We examined the BAL fluid of three children with asthma who were undergoing diagnostic flexible bronchoscopy. After two weeks of culture, one sample yielded colonies of adherent cells. These cells stained low for CD31, CD34 or CD45 and high for Stro-1, CD73, CD105, and CD166 (Figure 8A). Unlike normal human lung fibroblasts, but similar to pluripotent mesenchymal cells from human neonates [11], these cells could be differentiated along adipogenic, osteogenic and chondrogenic lines (Figure 8B). Together, these data suggest that pluripotent mesenchymal cells may be identified in the BAL of human asthma patients.

## Discussion

Mesenchymal stem cells are multipotent cells capable of differentiation into a select range of mesenchymal cell types influenced by their microenvironment. They are identified by their cell surface markers, high proliferation rate, ability to form colonies and differentiation potential [37]. More recent work shows that mesenchymal stem cells are not after all uniform, and that pluripotent mesenchymal cells may be separated into a hierarchy with differential self-renewal potential and multipotency [16].

Using cell sorting, we isolated a population of CD34- and CD45-negative, Stro-1-positive, high CD73- and CD105-positive cells. In humans, Stro-1 is almost exclusively found on bone marrow-derived MSCs [33]. Stro-1 has been described in bone marrow-derived stem cells in the mouse [34]. CD73 (SH3/SH4) and CD105 (SH2/endoglin) are localized at the plasma membrane of bone marrow mesenchymal stromal cells [36]. The CD34- and CD45-negative, Stro-1-positive, high CD73- and CD105-positive cells grew on plastic, demonstrated ample colony-forming potential, and were capable of differentiation to osteogenic, adipogenic, and myogenic phenotypes. Taken together, these data suggest that OVA-sensitization and -challenge increases the population of MSCs in the lung.

While we did not determine the precise function of MSCs in allergic airways disease, we consider two possibilities to be most likely. First, MSCs could constitute a source of airway myofibroblasts, leading to increased airway smooth muscle mass. Patients with severe asthma also demonstrate an accumulation of myofibroblasts in the airway subepithelium [19-21]. These alterations may extend beyond the central airways to the distal airways and lung parenchyma [23]. After allergen challenge, myofibroblasts in the subepithelium of the asthmatic airway may migrate to the smooth muscle layer [22]. Previous studies have noted increased airway smooth muscle cell number in fatal and non-fatal asthma [17,18], as well as OVA-sensitized and -challenged mice [38]. Mesenchymal stem cells generally express low levels of α-smooth muscle actin, but may express high levels of this contractile protein upon TGF-β treatment [11]. In multipotent stem cells from tracheal aspirates of premature infants, autocrine production of TGF-β further drives myofibroblastic differentiation,[13]. Finally, sorted lung MSCs were enriched for many transcripts which could play a role in myofibroblast differentiation and fibrosis including those encoding CD105/endoglin, part of the TGF-β receptor complex; the TGF-β family protein Gdf11 [39,40]; the TGF-β antagonists Gremlin, Lefty1 and BMP-binding endothelial regulator [41,42]; SMADs 2 and 3; NFATc3, a transcription factor involved in TGF-β1 signaling in airway smooth muscle [43]; and osteopontin which has been shown to be required for TGF-β expression and lung fibrosis in bleomycin- [44] and OVA-treated mice [45].

Another possible function of resident multipotent stromal cells is immunomodulation. Lung-resident mesenchymal stem cells from human lung allografts inhibit T cell proliferation *in vitro *[46]. Intravenously-administered murine mesenchymal stem cells derived from plastic-adherent bone marrow cells protect against bleomycin lung injury through the expression of interleukin-1 receptor antagonist [47-49]. In addition, intratracheal pluripotent mesenchymal stem cells have been shown to decrease endotoxin-induced lung injury [50]. In the present study, sorted lung MSCs were enriched in transcripts encoding the pro-inflammatory IL-6 family member cardiotrophin. This cytokine also is involved in ASM hypertrophy [43]. In addition, cultured lung multipotent stromal cells also produced MCP-1/CCL2 and KC/CXCL1, cytokines that would be expected to increase airway inflammation. Several members of the prolactin 2c gene family were greatly enriched in lung multipotent stromal cells. While humans possess only one prolactin gene used for both endocrine and paracrine functions, the prolactins are a multigene family in mice [51,52]. The prolactin-like peptides, also referred to as proliferins, have been implicated in wound-healing [53], angiogenesis [54] and B cell tolerance [55].

We successfully isolated MSCs from a patient with asthma, proof of concept that MSCs may be isolated from the BAL of these patients. As noted above, multipotent mesenchymal stem cells have been isolated from the tracheal aspirates of premature infants undergoing mechanical ventilation for respiratory distress [11] and the bronchoalveolar lavage of patients who have undergone lung transplantation [12]. In the latter studies, cells were grown from colonies identified 7-21 days after initial plating on plastic [11,12]. In the present study, MSCs were identified by flow cytometry after only 5 days of adherence to plastic, and by fluorescence microscopy at the time of allergen challenge. Together, these data provide stronger evidence that multipotent mesenchymal cells are present in the lung *in vivo. *However, at this time it is premature to say whether MSCs play a pathogenic role in human asthma.

In summary, we have found that allergen-sensitization and -challenge leads to the emergence of a lung cell population with the surface markers, differentiation potential and clonogenic potential of multipotent stromal cells. Further animal studies will be required to determine the physiologic role of these cells. Finally, additional work is needed to determine whether lung MSCs play a role in human asthma.

## Competing interests

The authors declare that they have no competing interests.

## Authors' contributions

JKB contributed aspects of all experimental designs, analyses and manuscript preparation; APP contributed to conceptual analyses of experiments; PDB contributed to conceptual analyses of gene chip experiments; MJL performed tissue processing, sectioning, and immunostaining; AEB assisted in developing differentiation assays; JL performed Western analyses; AMG performed ELISA and multiplex analyses; MBH contributed to conceptual design and analyses of experiments, manuscript preparation, and funding. All authors read and approved the final manuscript.

## Supplementary Material

Additional file 1 **Additional selected genes upregulated in sorted lung MSCs relative to lung fibroblasts**. This file contains a Table, Table S1, listing additional genes upregulated in sorted lung MSCs relative to total lung fibroblasts Click here for file
