# Supplementary material for: Ovalbumin sensitization and challenge increases the number of lung cells possessing a mesenchymal stromal cell phenotype
Source: Respir Res. 2010 Sep 21;11(1):127. doi: 10.1186/1465-9921-11-127 (PMC2949728; doi:10.1186/1465-9921-11-127)
Supplement: Additional file 1 — Additional selected genes upregulated in sorted lung MSCs relative to lung fibroblasts. This file contains a Table, Table S1, listing additional genes upregulated in sorted lung MSCs relative to total lung fibroblasts [file 1465-9921-11-127-S1.DOC]

***Table S1*** *Additional selected genes upregulated in sorted lung MSCs relative to lung fibroblasts*

| *Tyr Kinase receptors and receptor-linked* |  |  |  |  |
| --- | --- | --- | --- | --- |
| *Pik3r1* | 1700 | 276 | 6.16 | 6.29E-06 |
| *Igf1r* | 638 | 266 | 2.40 | 0.000812 |
| *Frk* | 176 | 44.7 | 3.94 | 0.00119 |
| *Epha2* | 105 | 24.9 | 4.22 | 0.00262 |
| *Erbb2* | 124 | 62.3 | 1.99 | 0.00896 |
| *Grb14* | 298 | 140 | 2.13 | 0.0142 |
| *Map2k2* | 1170 | 809 | 1.45 | 0.0158 |
| *Kras* | 681 | 452 | 1.51 | 0.0213 |
| *Rras2* | 767 | 269 | 2.85 | 0.0245 |
| *Sos2* | 118 | 59.8 | 1.97 | 0.025 |
| *Pdpk1* | 456 | 246 | 1.85 | 0.0286 |
| *Erbb2ip* | 180 | 90.4 | 1.99 | 0.0331 |
| *Eps8* | 581 | 145 | 4.01 | 0.0373 |
| *Epha7* | 30.6 | 5.3 | 5.77 | 0.0402 |
|  |  |  |  |  |
| *Cytokine and JAK-STAT signaling* |  |  |  |  |
| *Socs5* | 795 | 170 | 4.68 | 0.00016 |
| *Spred1* | 1280 | 678 | 1.89 | 0.00206 |
| *Stat6* | 87.1 | 26.9 | 3.24 | 0.00946 |
| *Socs6* | 252 | 111 | 2.27 | 0.0221 |
| *Il17rd* | 68.3 | 26.9 | 2.54 | 0.0277 |
|  |  |  |  |  |
| *TNF signaling* |  |  |  |  |
| *Traf2* | 166 | 33.6 | 4.94 | 7.09E-07 |
| *Ripk1* | 629 | 232 | 2.71 | 1.90E-05 |
| *Nkrf* | 308 | 94.7 | 3.25 | 2.90E-05 |
| *Traf4* | 334 | 222 | 1.50 | 0.000501 |
| *Tnfrsf12a* | 2460 | 416 | 5.91 | 0.00259 |
| *Nfkb1* | 422 | 316 | 1.34 | 0.00499 |
| *Tnik* | 57.4 | 15.1 | 3.80 | 0.0211 |
| *Nfkbib* | 41.8 | 14.5 | 2.88 | 0.039 |
|  |  |  |  |  |
| *GPCR Receptors* |  |  |  |  |
| *Avpr1a* | 649 | 236 | 2.75 | 0.000229 |
| *F2r* | 13600 | 8480 | 1.60 | 0.0188 |
| *P2ry5* | 749 | 459 | 1.63 | 0.0229 |

Abbreviations: *Pik3r1*, phosphatidylinositol 3-kinase, regulatory subunit, polypeptide 1 (p85 alpha);  *Igf1r*, insulin-like growth factor I receptor; *Frk*, fyn-related kinase; *Epha2*, Eph receptor A2; *Erbb2*, erythroblastic leukemia viral oncogene homolog 2; *Grb14*, growth factor receptor bound protein 14; *Map2k2*, mitogen activated protein kinase kinase 2; *Kras*, Kirsten rat sarcoma viral oncogene homolog; *Rras2*, RAS viral (r-ras) oncogene homolog 2; *Sos2*, Son of sevenless homolog 2 (Drosophila); *Pdpk1*, 3-phosphoinositide dependent protein kinase-1; *Erbb2ip*, Erbb2 interacting protein; *Eps8*, epidermal growth factor receptor pathway substrate 8; *Epha7*, Eph receptor A; *Socs5*, suppressor of cytokine signaling 5; *Spred1*, sprouty protein with EVH-1 domain 1, related sequence; *Stat6*, signal transducer and activator of transcription 6; *Socs6*, suppressor of cytokine signaling 6; Il17rd, encoding interleukin 17 receptor D; *Traf2*, Tnf receptor-associated factor 2; *Ripk1*, receptor (TNFRSF)-interacting serine-threonine kinase 1; *Nkrf*, NF-kappaB repressing factor; *Traf4*, Tnf receptor associated factor 4; *Tnfrsf12a*, tumor necrosis factor receptor superfamily, member 12a; *Nfkb1*, nuclear factor of kappa light chain gene enhancer in B-cells 1, p105; *Tnik*, TRAF2 and NCK interacting kinase; *Nfkbib*, nuclear factor of kappa light chain gene enhancer in B-cells inhibitor, beta; *Avpr1a*, arginine vasopressin receptor 1A; *F2r*, coagulation factor II (thrombin) receptor; *P2ry5*, purinergic receptor P2Y, G-protein coupled, 5.
